# Prostatic tissue: an unexpected finding in a mature ovarian teratoma: Case report and systematic literature review

**DOI:** 10.1007/s00404-021-06245-x

**Published:** 2021-10-07

**Authors:** Irene Pecorella, Maria Luisa Framarino dei Malatesta, Lucia Riganelli, Gaia Ciardi, Maria Grazia Porpora

**Affiliations:** 1 Department of Radiological, Oncological and Anatomical Pathology Sciences, University of Rome “Sapienza”, Viale Regina Elena 324, 00161 Rome, Italy; 2 Department of Gynecological and Urological Science, University of Rome “Sapienza”, Viale Regina Elena 324, 00161 Rome, Italy

**Keywords:** Ovarian teratoma, Prostatic tissue, Heterotopic tissue, PSA, AR, Immunohistochemistry

## Abstract

**Purpose:**

Prostatic tissue in an ovarian teratoma is an unusual finding, whose initiation in a 46, XX karyotype tissue is yet to be clarified. We present a case from our files and review the literature for this intriguing finding.

**Methods:**

Unstained histology sections of the ovarian teratoma containing prostatic tissue were evaluated using immunohistochemistry for PSA and androgen receptor.

**Results:**

Both PSA and androgen receptor immunostainings were positive in the prostatic tissue. From the literature review, it appears that most of the patients (74%) with similar findings were either pregnant or experiencing a miscarriage, menopausal or infertile at presentation, showing that an imbalanced hormone status is frequently associated with the presence of male structures in ovarian teratomas.

## Introduction

Mature cystic teratomas (dermoid cysts) are one of the most common benign ovarian neoplasms, accounting for 10–20% of all ovarian tumours. They arise from a single ovarian germ cell via parthenogenesis, following the first meiotic division. Molecular genetic analysis has shown that mature ovarian teratomas are usually homozygous for polymorphic markers [1], indicating that they most often derive from a germ cell that has completed meiosis I but not meiosis II, a conclusion supported by cytogenetic analysis [2].

They usually contain well-differentiated tissue from all three germ layers, that often duplicates the relationships seen in normal organs. As mature ovarian teratomas are diploid, and cytogenetic study demonstrates that they almost always have a normal 46, XX karyotype [3, 4], male sex organ structures are not an expected finding.

We report a case of benign ovarian teratoma which contained foci of mature prostatic tissue and review the literature cases.

## Case report

A 29-year-old woman presented with lower abdominal pain. No other complaint was reported and she had no evidence of virilisation. Ultrasound scans revealed a partially cystic ovarian right mass and a normal left ovary. Serum levels of sex hormones (testosterone, prolactine, LH, FSH) were within normal ranges. A 6-cm diameter right ovarian mass was removed laparoscopically. Cut sections showed a smooth-walled unilocular cyst filled with sebaceous material, and abundant blond hair. Focal thickenings of the cystic wall displayed a hard consistency.

**Fig. 1:**
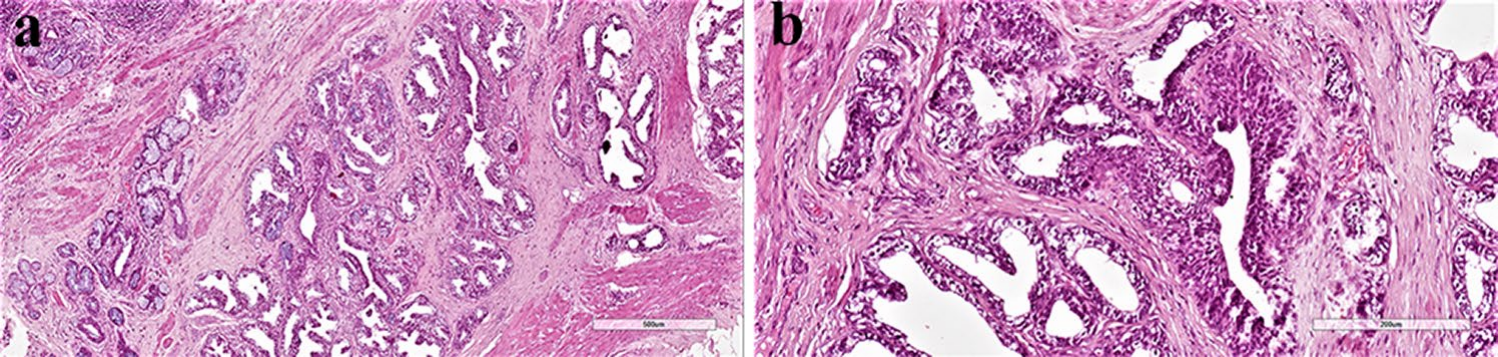
**a** Hematoxylin–eosin stained tissue sections of the ovarian teratoma showing prostatic acini (original magnification 20 ×). **b** At higher magnification, a duct lined by urothelium can be observed original magnification 200 ×)

**Fig. 2:**
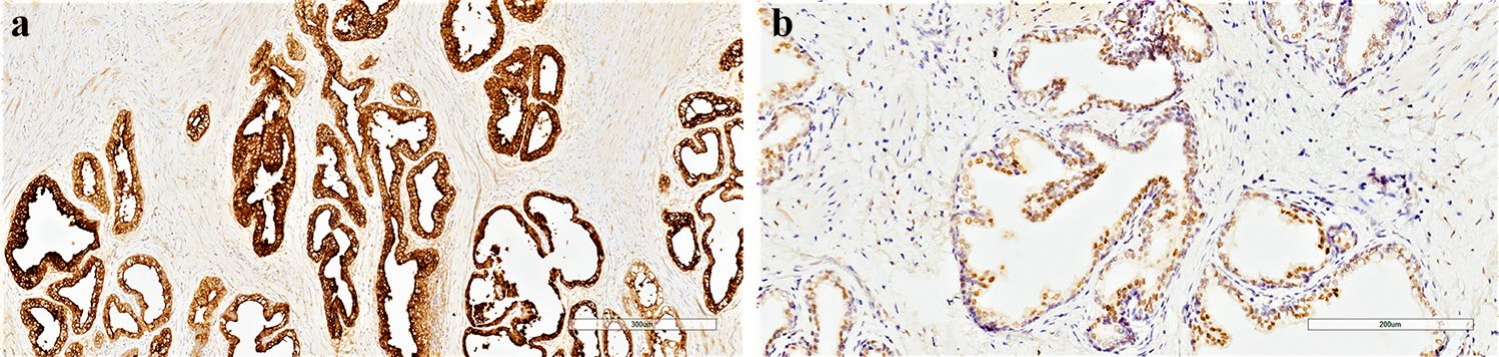
Immunoperoxidase stain shows strong and diffuse cytoplasmic positivity of the glandular epithelium with antibody to prostate-specific antigen (**a**) and nuclear positivity for androgen receptors (**b**) (original magnification 100 × , and 200 × , respectively)

Microscopically, the haematoxylin–eosin stained sections showed a typical mature ovarian teratoma with skin and cutaneous adnexal structures, fat, cartilage, respiratory and colonic mucosa. A focus of prostatic gland tissue, measuring 0.5 cm in diameter, was present next to the colonic mucosa and contained a central urothelium-lined duct (Fig. 1a, b). The prostatic glands demonstrated a simple branching pattern with well-developed terminal acinar tissue lined by cuboidal to columnar cells with pale cytoplasm (Fig. 1a, b). Basal cells were focally appreciable. Prostatic acini were surrounded by a fibromuscular stroma, reminiscent of the normal prostate. Sections of the remaining ovarian tissue failed to show nests of luteinized stromal cells, which appear as polygonal cells with abundant eosinophilic, often vacuolated, cytoplasm and provided with a central nucleus. Immunohistochemical staining was performed using a rabbit monoclonal anti-PSA antibody, clone EP1588Y (diluted at 1:100, Thermo Scientific LabVision, Italy) and a mouse monoclonal anti-androgen receptors (AR), clone SPM335, (diluted at 1:50, Abnova™, Italy) as primary antibodies on formalin-fixed, paraffin-embedded tissue sections. An immunohistochemical staining kit (Invitrogen Histostain^®^-SP, ThermoFisher), was subsequently used according to the manufacturer’s description. PSA immunostaining was positive in the cytoplasm of the lining cells (Fig. 2a). Antibody against AR depicted the nuclei of the prostatic acini, but not the surrounding ovarian stroma (Fig. 2b).

Written informed consent for patient information and images to be published was provided by the patient.

## Discussion

Ovarian teratoma can occur in persons of any age, although they are diagnosed most commonly during the reproductive years, with a median age of 35 yrs at presentation [5]. Only about 20.5% of the instances are postmenopausal women [5]. These tumours are most often right-sided, bilateral in 8–14% of cases, and show a median size of 6 cm, with approximately 60% of them measuring 5–10 cm in diameter [5].

A spectrum of different types of tissues may be identified in mature teratomas. In a careful study of 100 cases [6], ectodermal structures were found in 100%, mesodermal in 93%, and endodermal in 71%. Skin and the related structures are detected in 100% of the specimens and the dominant mesodermal structures are bone and/or cartilage. The most common endodermal component consists of thyroid tissue. Other tissue components consist of respiratory and intestinal epithelia, muscle, mature nervous and connective tissue, and less frequently, and choroid plexus. Carinelli et al. reviewed 228 ovarian teratomas and showed a 14% incidence for intestinal epithelia (never of duodenal type, though), and 55% for respiratory epithelium. Transitional-type epithelium often occurred in association with mucinous epithelium [7]. Thymus, pancreas, kidney, lung, retina, breast, hypophysis are rarely found [8].

Prostatic tissue also infrequently occurs in ovarian teratoma and is even rarer in testicular teratoma [9].

Blackwell et al. in their microscopical review of 100 ovarian teratomas with multiple sections (10–15) found only a single case (1%) containing prostate tissue [6]. On the other hand, Vadmal et al. reported prostatic tissue in 12% of 25 carefully sampled ovarian teratomas, and claimed that this finding may be more common than currently reflected in literature [10]. Our systematic literature search yielded only 34 published cases (Table 1). An additional paper by Bertrand et al., which is often mentioned [11], corresponds in reality to a letter to the Editor of the journal with a comment on Brumback et al. reported case [12]. Therefore, despite Vadmal et al. statement [10], ovarian teratoma does not appear to commonly harbour prostatic tissue.

**Table 1:** Published cases of ovarian teratoma containing male structures

Ref.	Age (yrs)	Cyst ∅ (cm)	Side	Focus ∅ (cm)	Other findings in the prostatic tissue	Male accessory sexual structures and/or transitional epithelium	Associated tissues	Pt’s hormone status
[3]	33	na	L	na	PSA + , PAP + , Cam 5.2, CK 34bE12 in basal cells	Transitional epithelium, Cowper’s glands	Skin and appendages, neural and respiratory tissue	Infertile
	54	na	R	na	PSA + , PAP + , Cam 5.2, CK 34bE12 in basal cells	Transitional epithelium	Skin adnexa, fat, bone,	Nulliparous, menopause
[4]	17–38	4–7	2 R 2 L	0.2–1.9	46 XX or XXX in 2 cases PSA + AR focal in 1 of 2 tested	1 prostatic ADK Transitional epithelium in 2 cases	Lutheinised cells	2 normal 1 tubal pregnancy 1 infertility
[6]	na	na	na	na		na	na	na
[8]	20	4	L		PSA + , PAP +	Transitional epithelium	Skin and appendages, fat	Normal
[10]	23 71 33	10 9 8	na na na	1.5 1.2 2	PSA + , PAP + PSA + , PAP + PSA + , PAP +	Transitional epithelium in all 3 cases	Skin and appendages, bone, cartilage, neural tissue	na Menopause na
[12]	18	6	R	na	PSA + , PAP +	None	Skin and appendages, glial tissue, tooth	Therapeutic abortion
[13]	31	5	L	na	PSA + , PAP, cytok 7 (urethra), AMACR− (PIN)	High-grade PIN, urethra	Skin and appendages, intestinal and respiratory mucosa, cartilage	Normal
	20	8	L	0.8			Skin and appendages, intestinal and respiratory epithelium, cartilage, bone	Dysmenorrhea
[14]	32	11	L	1.2	PSA +	Transitional epithelium (bladder?), prostatic adenocarcinoma	Skin and appendages, tooth, bone	Obesity, diabetes type 2
[15]	30	5	R		PSA + , PAP +	Cowper’s glands, and seminal vesicles	Skin and appendages, respiratory and intestinal epithelia, cartilage, muscle, and nervous and connective tissue	Hashimoto thyroiditis
[16]	50	nr	L	1.5	Double Barr bodies in 15% of teratoma cells	Transitional epithelium	Skin and appendages, glia, fat, smooth muscle, respiratory tissue, lutheinised cells	na
[17]	20	7.5	L	0.8	PSA + , PAP + , 34bE12 in basal cells	Transitional epithelium	Shin and appendages, respiratory, gastric, colonic mucosa, thyroid, glia, smooth muscle, bone, cartilage, tooth Lutheinised cells	Dysmenorrhea
[18]	15	13	R	na	PSA + , PAP +	Cowper’s glands, transitional epithelium, corpora amilacea	Gastric, colonic, respiratory tissue, smooth muscle, thyroid	Normal hormone levels
[19]	73	11.5	L	na	PSA + , PAP + , Cam 5.2 +	Transitional epithelium Cowper’s glands	Skin and appendages, intestinal and respiratory epithelium	Menopause
[20]	37	na	na	na	PSA + , PAP +	Transitional epithelium	Skin and appendages, adipose and smooth muscle tissue, nerves	na
	30	na	na	na	PSA + , PAP +	na	Bone and bone marrow, fat, sebaceous glands	na
[21]	40	8	L		PSA + , 34bE12 in basal cells, urothelium	Transitional epithelium	Skin and appendages	Miscarriage
	30	9	L	1.2	PSA + , 34bE12 in basal cells, urothelium	Cowper’s glands, cavernous bodies and transitional epithelium	Skin and appendages, respiratory and intestinal epithelium, brain tissue, fat and smooth muscle tissue	Delivery
[22]	46	6	L	na	PSA + , PAP +	Transitional epithelium		Nulliparous (infertile?)
	51	7	R	na	PSA + , PAP +	Cowper’s glands, transitional epithelium	Skin, neural tissue and respiratory epithelium	Menopause
[23]	21	10	nr	nr	PSA + , PAP +	nr	Cartilage, skin and appendages, salivary glands	18 wks gestation
[24]	23	10	L	1.5	PSA + , CK 34bE12 in basal cells	Transitional epithelium		Dysmenorrhea
[25]	56	2	L	0.5	PSA + , PAP + , CK 34bE12 in basal cells	Transitional epithelium	Connective tissue, smooth muscle tissue, fat, bone, respiratory and salivary epithelia	Menopause
[26]	30	6.3	L	1.6	PSA + , ER−, CK34βE12 + (basal cells, urothelium, Cowper’s mucinous glands), CD10 + luminal cells of prostatic glands	Urethral duct, Cowper’s glands	Skin and adnexa, tooth	Pregnant. Normal hormone levels
[27]	15	na	R	0.5	na	na	na	na
[28]	31	6	na	na	ER + , PR + . PSA + , PAP + , CK34βE12 + (basal cells)	None	Skin and appendages, respiratory mucosa, mucinous cystadenoma	Normal
[29]	22	10	R	na	PSA + , PAP + , citok 5/6 + in basal cells	Transitional epithelium, urethral duct	Skin and appendages, respiratory epithelium, salivary glands, cartilage	14 wks’ gestation
[30]	24	9	na	na	PSA +	Transitional epithelium	Skin and appendages, glia, neural tissue, gastrointestinal, bronchial epithelium, salivary glands, bone, cartilage, fat, smooth and striated muscle	Normal
Our case	29	6	R	0.3		Transitional epithelium	Skin and appendages, fat, cartilage, respiratory and colonic tissue	Na

*L* left; *R* right; *na* not available; *np* not performed; nr: not reported; *PAP* prostate acid phosphatase; *PSAP* prostate specific acid phosphatase; *PSA* prostate specific antigen

Review of the published data showed that the mean age of patients at presentation was 33.6 yrs, in accord with the usual age for ovarian mature teratoma. None of the reported cases showed signs of virilisation. The mean diameter of the cyst was 7.8 cm, also in keeping with the findings in usual ovarian teratomas. As to laterality, however, 61.5% of cases was left-sided (16 out of 26 recorded cases). Premalignant or malignant transformation was observed in three instances (8.8%) [4, 13, 14].

Urothelial structures were always present next to the prostatic glands. This is an interesting association as, overall, transitional type epithelium is rare in ovarian teratoma. The constant association of fetal urothelial epithelium can be explained by the endodermal derivation of the prostate induced by a urethral bud which arises from the urogenital sinus. Experimental work has shown that the urogenital sinus mesenchyme (UGM) instructively induces bladder and urethral epithelium to form prostate [31]. The urethral bud responds through nuclear androgen receptors that are activated by testosterone or dihydrotestosterone. UGM is capable to transform testosterone into dihydrotestosterone. Androgens are important for stimulating prostate ductal growth and branching morphogenesis as well as for establishing functional differentiation of luminal secretory epithelial cells. UGM receptors are required for establishing prostate identity; epithelial receptors are required for establishing secretory function in the epithelium. Grafting and tissue recombination experiments have shown that paracrine signals from the developing UGM also direct lobe-specific identity in the juxtaposed epithelium [32]. Epithelial potential to respond to paracrine signals from the UGM by forming prostate is restricted to endodermal epithelia with similar embryonic origin to the prostate. Instructive paracrine signals from the prostatic epithelium, in turn, induce and pattern the surrounding smooth muscle. Knowledge on the normal development of the prostate points, therefore, to the crucial role of androgens. Androgens are required and sufficient to establish prostate identity in the urogenital sinus. However, in addition to androgens, prostatic development is very sensitive to levels of estrogenic compounds. It is possible that the formation of prostatic tissue in ovarian teratoma might be the result of transient hormone imbalance in the patients. Indeed, out of 27 recorded cases, 5 patients were menopausal, 7 were pregnant or had a miscarriage, 6 were infertile or dysmenorrheal, 1 was obese and suffered from diabetes type 2, 1 had Hashimoto’s thyroiditis. This shows that 74% of this study population had some hormone imbalance. How the hormonal imbalance may affect the UGM and induce prostatic tissue in an ovarian teratoma remains to be elucidated. Yet, the expression of PSA in the prostatic epithelium of the present case demonstrates that the luminal epithelial cells were functionally differentiated toward prostate-specific proteins secretion, due to the action of androgens.

Table 1 shows that Cowder’s bulbo-urethral glands, which also derive from the urogenital sinus, were also rather frequently observed (25%). On the other hand, structures from the central zone of the prostate, as well as male accessory sex glands, such as rete testis, epididimys, vas deferens, seminal vesicles and ejaculatory ducts, which develop embryologically from the mesodermal Wolffian ducts were only exceptionally described (Table 1).

The determination and initiation of prostatic development in the human and rodent foetus are initially entirely dependent upon AR; however, later on, prostatic glands budding can continue to a large degree in the absence of testosterone due to irreversible commitment of the tissue.

In ovarian teratoma, the hormonal androgen microenvironment could also be provided by the tumour itself. Three investigators have identified luteinized cells in the ovarian stroma and indicated these as the source of the androgen stimulation [4, Table 1]. However, most authors have not detected such morphological changes in the ovarian stroma (Table 1) and we did not found it either in our patient’s specimen. It should be noted, however, that the cyst wall in the present teratoma contained only minimal residual cortical ovarian tissue, while luteinized stromal cells are most often located in the medulla. There, they can be recognized as medium-sized polygonal cells with abundant eosinophilic cytoplasm and prominent nucleolus in up to 13% of women under the age of 55 years.

Other possible androgenic stimuli may derive from ovarian hilar Leydig cells, or adrenal cortex.

Nonteratoid prostatic differentiation in the ovary is also a possibility. Other possible explanations for the presence of prostatic tissue in an ovarian teratoma include development from genetic material derived from the paternal chromosomes that failed to be inactivated during parthenogenesis.

The endodermal origin of the peripheral prostate explains the close relationship of the prostatic tissue with the colonic mucosa in our case and other similarly reported close contacts. In most published cases, staining with prostatic markers was focal rather than diffuse and was always confined to the glandular elements. In all the benign cases tested, staining with 34bE12 highlighted the basal cell layer, demonstrating normal morphogenesis of the prostatic tissue. The glandular cells were negative with estrogen receptor and progesterone receptor. Similar to normal prostatic tissue, the glands in teratoma were characteristically CD10 positive.

In conclusion, prostatic tissue in ovarian teratomas is a rare and paradoxical finding, which could possibly be explained by development from urogenital sinus in patients with hormone imbalance.

## Data Availability

Available upon reasonable request.
